# The Influence of Subclinical Neck Pain on Neurophysiological and Behavioral Measures of Multisensory Integration

**DOI:** 10.3390/brainsci9120362

**Published:** 2019-12-09

**Authors:** Antonia M. Karellas, Paul Yielder, James J. Burkitt, Heather S. McCracken, Bernadette A. Murphy

**Affiliations:** 1 Faculty of Health Sciences, University of Ontario Institute of Technology, Oshawa, ON L1G 0C5, Canada; antonia.karellas@ontariotechu.net (A.M.K.); paul.yielder@uoit.ca (P.Y.); jiemburkitt@gmail.com (J.J.B.); heather.mccracken@ontariotechu.net (H.S.M.); 2 School of Medicine, Deakin University, Geelong, VIC 3216, Australia

**Keywords:** electroencephalography (EEG), event-related potential (ERP), multisensory integration (MSI), somatosensory integration (SMI), subclinical neck pain (SCNP)

## Abstract

Multisensory integration (MSI) is necessary for the efficient execution of many everyday tasks. Alterations in sensorimotor integration (SMI) have been observed in individuals with subclinical neck pain (SCNP). Altered audiovisual MSI has previously been demonstrated in this population using performance measures, such as reaction time. However, neurophysiological techniques have not been combined with performance measures in the SCNP population to determine differences in neural processing that may contribute to these behavioral characteristics. Electroencephalography (EEG) event-related potentials (ERPs) have been successfully used in recent MSI studies to show differences in neural processing between different clinical populations. This study combined behavioral and ERP measures to characterize MSI differences between healthy and SCNP groups. EEG was recorded as 24 participants performed 8 blocks of a simple reaction time (RT) MSI task, with each block consisting of 34 auditory (A), visual (V), and audiovisual (AV) trials. Participants responded to the stimuli by pressing a response key. Both groups responded fastest to the AV condition. The healthy group demonstrated significantly faster RTs for the AV and V conditions. There were significant group differences in neural activity from 100–140 ms post-stimulus onset, with the control group demonstrating greater MSI. Differences in brain activity and RT between individuals with SCNP and a control group indicate neurophysiological alterations in how individuals with SCNP process audiovisual stimuli. This suggests that SCNP alters MSI. This study presents novel EEG findings that demonstrate MSI differences in a group of individuals with SCNP.

## 1. Introduction

Today’s culture is immersed in technology. Throughout the day, several hours are spent on desktops, laptops, and phones. This promotes sustaining awkward uncomfortable postures throughout the day [1,2]. Professionals, such as office workers, who maintain these static postures are therefore susceptible to experiencing symptoms of neck pain [1,2]. Chronic neck pain and muscle fatigue affect approximately 30–50% of the general population [3]. Recurrent, untreated neck pain of mild to moderate severity is termed subclinical neck pain (SCNP) [4]. Given that SCNP is common, it is surprising that little is known about the long-term consequences of altered neck sensory input on brain processing. SCNP’s impact on musculoskeletal and neurophysiological systems is an emerging topic of great interest that has provoked researchers to investigate the implications of SCNP on the somatosensory system. Abnormal recruitment patterns of neck muscles in the SCNP population can lead to altered movement patterns of the upper limb [5] and altered upper limb joint position sense [6], indicating altered sensorimotor integration (SMI).

SMI is the process by which the central nervous system (CNS) integrates sensory information from different body parts and formulates appropriate motor responses [7]. Learning new motor skills properly and performing tasks without making errors is reliant on proper functioning of the sensorimotor system. Chronic changes in sensory input from the neck due to neck pain is known to lead to SMI [8]. This changes the way that sensory input from the upper limb is processed, by inducing plastic changes in the CNS [5]. Maladaptive plasticity resulting from SCNP and stiffness distorts SMI, and consequently, elicited motor responses do not accurately correspond to the sensory input required for accurate task performance [9]. Musculoskeletal disorders therefore impact CNS processing and integration of sensory information [9]. The recurrent component of SCNP allows researchers to investigate the long-term neurophysiological changes resulting from altered afferent input from the neck. Importantly, those with SCNP can be tested on pain-free days, eliminating the possibility of the confounding effect of acute pain, which is known to affect both somatosensory evoked potential SEP amplitudes [10] and movement patterns [11].

Existing literature has demonstrated that individuals with SCNP experience altered joint position sense (JPS), which is defined as a person’s ability to perceive the position of his or her joints [6]. Individuals with SCNP demonstrate significantly reduced elbow JPS compared to their healthy counterparts [6]. This suggests that SCNP influences the processing of proprioceptive information from the brain. Additional research reveals decrements in motor learning and mental rotation resulting from neck pain [12,13]. If SCNP alters SMI, it is probable that it would alter multisensory integration (MSI) as well.

MSI is the process by which the brain processes and integrates multiple sensory inputs from different sensory modalities [14,15]. Effective MSI is an important aspect of many occupational and daily living tasks, such as driving, crossing the road, or learning in a classroom. Auditory (A) and visual (V) integration of information is essential for effective object recognition, communication, and overall exploration of the world [16]. Further, postural control is influenced by MSI of the vestibular, proprioceptive, and tactile sensory inputs as well [14,17]. According to Meredith and Stein [18], at the neural level, MSI is the statistically significant difference between the number of impulses evoked by presentation of unisensory stimuli independently versus the cross modal combination of these stimuli. MSI can therefore lead to a neuronal response that is enhanced or depressed when compared to unisensory responses [19,20].

The involvement of cortical and subcortical brain regions in MSI has also been demonstrated by existing literature. The superior colliculus is a subcortical brain structure that is abundant in multisensory neurons, which integrate signals from different sensory modalities [20,21]. These modalities may be A, V, or somatosensory in nature [22]. The parietal region is known for its involvement in MSI as well [19,23,24]. MSI has been shown to occur in the superior region of this lobe [25], as well as in the intraparietal sulcus [19].

It is suggested that alterations in afferent input results in the reweighting of sensory input, meaning that adjustments are made in terms of how much different senses contribute to perception [8]. The reweighting of MSI has been assessed in the elderly population, using behavioral methods [26]. According to Eikema et al. [26], fall risk increases in the elderly population as a result of reweighing of multisensory input. In the elderly population, there is a reduction in visual and proprioceptive cues, which contributes to the great differences in posture between the elderly and younger populations [14]. Inverse effectiveness is thought to play a role in levels of multisensory enhancement seen in different populations. This theory suggests that in circumstances where responses to multisensory stimuli are weak, enhancements in MSI may occur [18]. Such an effect was seen in a study conducted by Hairston et al. [27], which investigated whether differences in MSI exist in localizing V, A, or AV targets under normal visual conditions and with induced myopia. Interestingly, the induced myopia condition improved localization skills for the multisensory targets, while in the normal condition, localization skills for the unisensory conditions did not significantly differ from the multisensory condition [27]. If neck pain creates decrements in unisensory processing of auditory or visual stimuli and multisensory enhancement is still evident in this population, inverse effectiveness may potentially be responsible for this. Alternately, because neck pain is known to alter upper limb proprioception, as indicted by decreased awareness of joint position sense [6], the fact that upper limb proprioception is less reliable in this population could potentially lead to increased reliance on AV inputs for adequate MSI.

Current literature has measured MSI behaviorally, by analyzing reaction time (RT) and accuracy, as study participants perform a task involving multisensory stimuli [15,28,29,30,31]. A study by Farid et al. [28] used a two-alternative forced-choice discrimination task modified from Laurienti et al. [30] to assess differences in RT and accuracy between individuals with no recent history of neck pain and an SCNP group. Results of this study demonstrated slower response times for visual and audiovisual tasks in the SCNP group, suggesting that altered afferent input from the neck may influence MSI in the SCNP population. To understand the neurological factors that contribute to MSI in SCNP, a neurophysiological technique combined with behavioral measures has the potential to demonstrate differences in neural responses to multisensory stimuli between populations.

Electroencephalography (EEG) is a neurophysiological technique that measures electrical activity generated by underlying brain regions from the scalp [32]. EEG has been effectively used as a tool to accomplish this in children and adolescents with autism spectrum disorder [23,24]. Brandwein and colleagues [23,24,33] combined behavioral measures with high-density electrical mapping of event-related potentials (ERPs) to determine the development of MSI from middle childhood to early adulthood. These researchers used a simple RT task to assess differences in simple response times and neurophysiological measures between populations. The methodology of this study is reliable and replicable. It also enables the initial integration to audiovisual (AV) stimuli to be measured, which is a critical first step prior to progressing to more advanced paradigms.

Using EEG, it is a common practice for neuronal responses to multisensory conditions to be investigated by directly comparing them to the sum of the neural responses to the unisensory conditions, which is based upon the principle of superposition of electrical fields [19,34,35,36,37]. This method of MSI quantification is based upon two waveforms. The first waveform is known as a multisensory waveform, representing the sensory processing occurring in response to the multisensory condition. The second waveform is known as the sum waveform, which is the summation of sensory responses to the two unisensory conditions; with respect to this research, this involved the summation of the unisensory A + V activity. This technique has been applied to a variety of studies which investigated differences in multisensory integration occurring between different populations [23,34,36,38]. Any significant divergence between the additive event-related potentials (ERPs) of the unisensory conditions and the multisensory ERP is indicative of MSI occurring [19,34,35,36,37]. Electrical fields generated by neurons are detected and measured by ERP recordings. Due to the fact that the electrical fields sum linearly, it is acceptable to predict that with synchronously firing neurons, the ERP response would be the linear sum of the presented unisensory components [37]. If the multisensory neural response is greater than the sum of the unisensory modalities’ neural activity, superadditive responses have occurred. Alternatively, if the multisensory response is lower than the sum of unisensory responses, the multisensory response is subadditive [19]. With both scenarios, multisensory enhancement has occurred. Superadditivity classifies the degree to which enhancement has occurred, with superadditivity indicating greater enhancement as opposed to subadditivity [20].

The current study investigates differences in MSI that may exist between individuals with SCNP and individuals with no recent history of neck pain. This was completed by recording electrical brain activity using EEG, as participants perform a simple RT multisensory task, as has been successful in previous research [23,24]. This builds on existing literature by improving knowledge of the long-term neurological consequences of neck pain on the multisensory system. There are two competing hypotheses for this research, which are that: (1) Individuals with neck pain will demonstrate slower RT, due to reductions in the degree of multisensory enhancement, as seen in the literature [28]. This will be accompanied by differences in the level of brain activity occurring in those with SCNP. ERP amplitudes will potentially be different in those with SCNP compared to the healthy control group, with a smaller divergence occurring between the multisensory and sum ERPs. (2) In line with the inverse effectiveness theory, individuals with neck pain will demonstrate increased reliance of AV inputs (MSI) due to worse proprioception [5,9], which makes proprioceptive inputs less reliable. Therefore, in this instance, there would be a greater divergence occurring between the multisensory and sum ERPs, which would indicate enhanced MSI.

## 2. Materials and Methods

### 2.1. Participants

Twenty-four participants with no known neurological conditions were recruited for this study (mean age: 21.5; range: 19–28). The control group (*n* = 12) consisted of individuals with no recent history of neck pain, while the experimental group (*n* = 12) comprised participants with SCNP. Four males and eight females were included in each group. Both groups were balanced according to hand dominance using the Edinburgh Handedness Inventory [39]. Informed consent was obtained from every participant and the study was approved by the ethics committee (REB #14686) at the University of Ontario Institute of Technology, and has therefore been performed following the ethical standards outlined by the Declaration of Helsinki.

Prior to beginning the experiment, participants completed preliminary screening to establish the absence of any medical or neurological conditions. The neck disability index (NDI) [40] and the Chronic Pain Grade Scale [41] were completed to assess pain levels and history of neck pain. A neck pain mini questionnaire was also completed to obtain an improved understanding of any previous injuries and occurrence of neck pain. Data collection only occurred on pain-free days for those with SCNP and these participants must not have received chiropractic treatment for at least 6 weeks prior to the measurement session.

### 2.2. Procedure

#### 2.2.1. Electroencephalography Set-up and Acquisition

The experiment session began with the set-up of the EEG system. The cap was carefully placed on the head in the anterior to posterior direction, meaning once the frontal electrodes were applied, the cap was pulled back over the rest of the head. The protocol for the placement and positioning of electrodes on the scalp is standardized according to the International 10/20 System, which was adopted in 1958 by the International Federation in Electroencephalography and Clinical Neurophysiology [42]. This standardized method divides the head into proportional distances from four main reference points [43]. The letters F, T, C, P, and O represent the region of the head that the electrodes are positioned over [32,43]. These regions are frontal, temporal, central, parietal, and occipital, respectively [43]. Electrodes with odd numbers are on the left side of the head, while the even numbered electrodes are found on the right side of the head. Smaller numbers are located closer to the midline. The cap was centered with the CZ electrode positioned in the center of the head, as required by the standardized technique. Measurement of the CZ position involves measuring half of the distance between the nasion and the inion, and half of the distance between the pre-auricular points. The vertex electrode was positioned where these two points intersect. A conductive gel was used to fill the small hole of each silver–silver chloride electrode by using a syringe. This gel filled the space between the electrode and the skin to lower impedance and to improve the overall quality of the signal.

EEG was continuously recorded using the ANT Neuro Imaging Waveguard TM 64 Lead EEG cap (ANT Neuro, The Netherlands) and Advanced Source Analysis™ software (Version 4.10.1). The signal was recorded at a sampling rate of 2048 Hz. The collected recording was referenced to the software’s “ref” electrode setting. The EEG amplifier used in this study was the TMSi REFA-8 amplifier (3.5 W-10VDC) (Oldenzaal, The Netherlands).

#### 2.2.2. Simple Reaction Time Multisensory Integration Task

**Figure 1:**
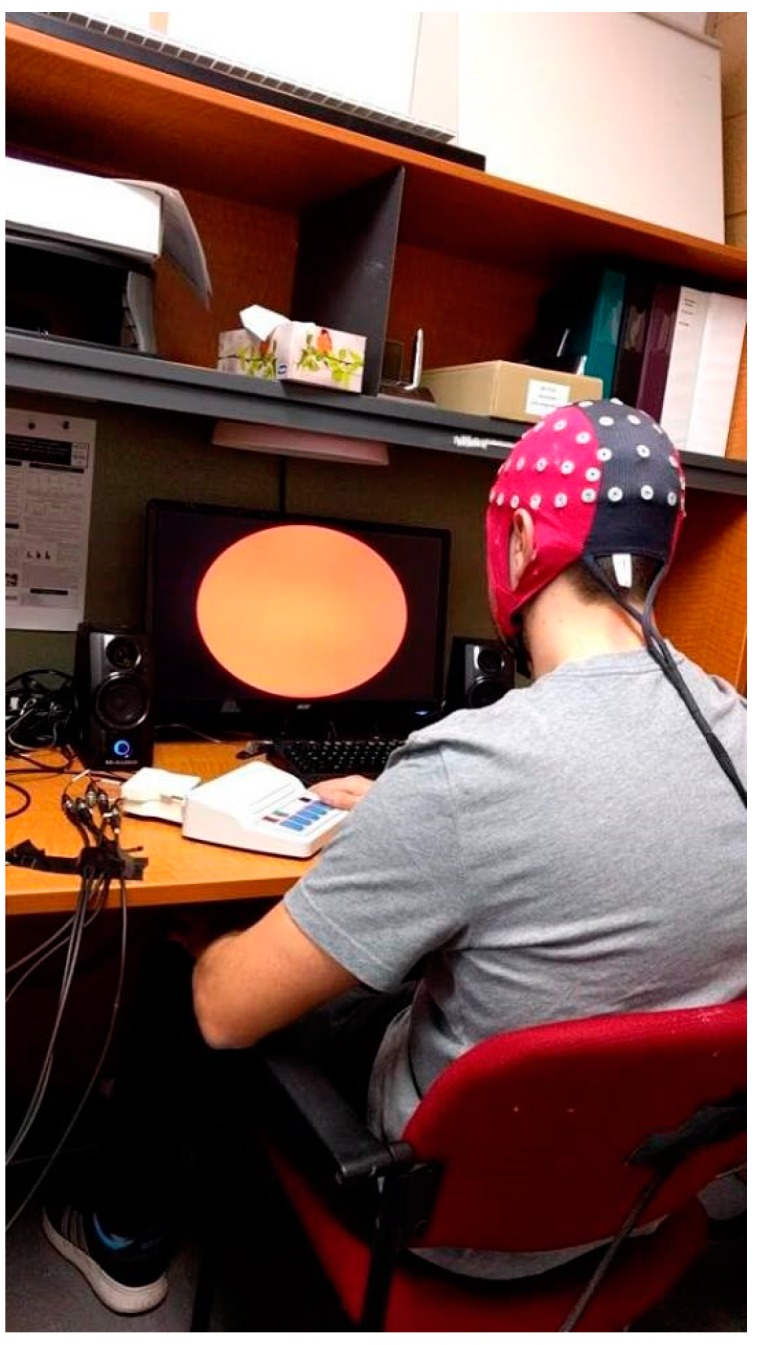
This image portrays the experiment set-up and the equipment used for data collection. The participant is seated directly in front of the monitor with their right thumb placed over the response key. The EEG recording takes place during task performance.

MSI was measured using a simple RT multisensory task, similar to that used by Brandwein and colleagues [23,24]. The paradigm utilized E-Prime^®^ 2.0 Professional software (Psychology Software Tools, Pennsylvania) on a Windows 8.1 desktop computer. Participants were comfortably seated and centered to the desktop computer. The experiment set-up is demonstrated in Figure 1. The task consisted of the randomized presentation of three stimulus conditions, which included a unisensory A, unisensory V, and multisensory AV condition. Each stimulus presented the color “red” through the use of a different modality. Specifically, two M-Audio Studiophile AV 20 speakers placed bilateral to the Acer monitor emitted the auditory female verbalization of the word red (duration ~180 ms). The visual stimulus was a visual representation of the color red, in the form of a red disk with a diameter of 30 cm appearing on the black screen for a duration of 60 ms. A three-dimensional (3D) gaming monitor (Acer GN246HL Bbid) was used to present this stimulus to the participant. The multisensory AV condition consisted of the simultaneous presentation of both unisensory conditions. A fixation point preceded each stimulus presentation and lasted for a randomized duration of 1000–3000 ms. The randomized interstimulus interval aimed to minimize responses driven by prediction and anticipation of stimulus onset, limiting the influence of anticipatory potentials to the ERP [44]. Therefore, maintained attention would be required for appropriate responses to each stimulus condition.

**Figure 2:**
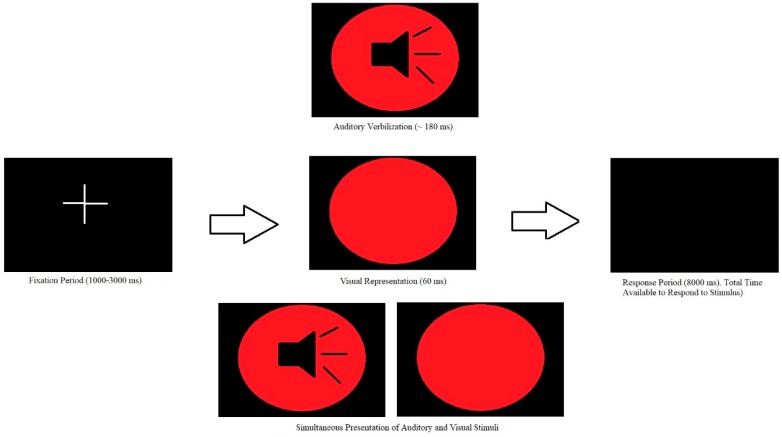
This figure displays the multisensory simple reaction time (RT) task. The stimulus presentation process is depicted above. Participants were randomly presented with an A, V, or AV stimulus, each of which were preceded by a fixation period with a randomized duration of 1000–3000 ms. Participants responded on a response key as soon as they saw the stimulus.

The task consisted of 8 blocks, with each block consisting of 34 A, 34 V, and 34 AV conditions [23,24]. Participants were instructed to respond as quickly as possible to each stimulus by pressing the response key on the Chronos^®^ multifunctional response and stimulus device placed in front of them using their right thumb. The Chronos^®^ device has millisecond accuracy. All participants kept their thumb resting on the response key for the duration of the experiment. Figure 2 serves as a visual representation of the task.

### 2.3. BehaviouralAnalysis

#### 2.3.1. Task Performance Analysis

Button press responses to the presentation of all stimulus conditions were recorded during task performance. RTs were only included if they fell within ±2 standard deviations of each participant’s average RT for their respective A, V, and AV condition This was done to ensure that trials where participants were not paying attention, and hence not performing the task, were not included in the final analysis. Differences in RT between conditions were compared using Tukey’s honestly significant different post-hoc tests.

RT performance was compared between groups for all stimulus conditions using a 2 GROUP (control, SCNP) by 3 STIMULUS CONDITION (V, A, AV) mixed factors analysis of variance (ANOVA), with repeated measures on the last factor. All statistical tests were performed using IBM SPSS^®^ Version 25 (Armonk, NY, USA) and for all analyses, alpha was set at *p* < 0.05. Post-hoc analysis (Tukey’s test) was used to decompose any significant main effects and interactions involving more than 2 means.

#### 2.3.2. The Race Model

Miller’s Race Model [31] was performed as an additional measure to establish whether any multisensory facilitation (faster RT for the multisensory stimulus) is due to the neural processing of multisensory stimuli, or is simply a result of the faster of the two independently processed unisensory stimuli triggering the response. The Race Model is calculated from a cumulative distribution function (CDF) by the joint probability of the unisensory condition responses. Violation of the Race Model, indicated by a greater probability for a response to the multisensory conditions compared to the joint probability of the unisensory conditions, indicates that neural integration of the stimuli has occurred, and results obtained are not due to the redundant nature of the stimulus [45].

The Race Model CDFs were calculated for each participant using the MATLAB algorithm developed by Ulrich et al. [45]. This algorithm outputted Race Model (i.e., indicting no multisensory integration) and multisensory CDFs for 10% quantiles of RTs falling within that bin of responses that occurred within that latency for each condition. Paired sample *t*-tests were performed independently for the control and SCNP conditions at the quantiles where the multisensory condition CDF was faster than the CDF predicted by the race model (i.e., the multisensory condition probability curve lying to the left of the race model probability curve). This was done to determine if group differences in RT, where the multisensory condition was faster, are due to multisensory integration or simply the faster of the two unisensory stimuli triggering the response.

### 2.4. Neurophysiological Analysis

#### 2.4.1. Artifact Removal

Advanced Source Analysis™ (ASA 4.10.1) software was used for processing and analysis. A low pass filter of 45 Hz and a high pass filter of 1.6 Hz were applied to the data. The filter steepness was set to 24 db/octave and 12 db/octave for the low and high pass filters, respectively. Following filtering, automatic artifact detection (±100 µV) was performed to ensure the removal of any remaining artifacts that may not have been removed by steps previously performed [24]. Then, 600 ms epochs were created to isolate brain activity from 100 ms pre-stimulus to 500 ms post-stimulus onset [24]. All epochs were sorted and averaged according to stimulus condition. This was done for each participant to generate grand average ERPs for the V, A, and AV stimuli. Group grand averages were created for the V, A, and AV conditions for the control and SCNP groups to compare overall brain activity between populations.

#### 2.4.2. EEG/Epochs Analysis Method 1

**Figure 3:**
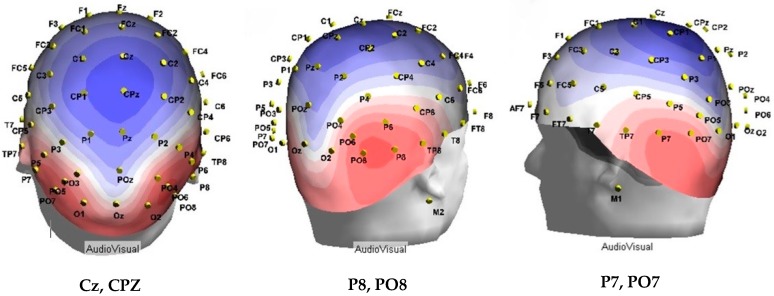
A visual representation of the most active brain regions between 100 and 140 ms post-stimulus presentation for all participants as revealed by topographical analysis.

Similar to previous studies [34,35,36], AV interactions were measured by comparing a grand average sum waveform with a grand average multisensory waveform combined across all participants at the brain regions showing greatest activity during task performance [23,24,34,35,36]. The sum waveform is the summation of the average unisensory condition waveforms (visual average ERP + auditory average ERP = sum ERP). The principle of superposition of electrical fields indicates that any significant dissimilarity between these waveforms is indicative of MSI, as this would mean that an interaction occurred between the unisensory stimuli when presented simultaneously in the multisensory condition [18]. This analysis was constrained to include only the electrodes that were most active during task performance at early pre-cognitive latencies of 0–200 ms [34,46]. Reasons for this are (1) integration effects can be expected to occur during the earlier stages of sensory processing, as indicated by evidence of subcortical interactions in animal studies [35]; and (2) auditory and visual ERPs reflect neural activity that is modality specific below 200 ms [35]. Specifically, using the ASA software, a voltage map was created which illustrated electrodes and latencies of greatest positive and negative electrical activity. Topographical analysis for this study allowed the researcher to identify the time regions where the greatest neural activity occurred with millisecond accuracy. Figure 3 depicts the most active brain regions with the densest blue (−µV) and red (+µV) colors. Overall, this procedure was done to ensure unbiased selection when choosing the brain regions in which to assess multisensory interactions between the groups. Using MATLAB R2017a software ^®^ (Natick, MA, USA), grand average sum and grand average multisensory waveforms were generated for each participant across every time bin. A grand average ERP was obtained for each time bin, across the group of electrodes that was shown to be most active, as indicated by the topographical analysis.

Topographical analysis revealed the greatest level of brain activity occurred between 100 and 140 ms post-stimulus onset. This was indicated by the dense red and blue colors. During this time frame, the MSI head model demonstrated that the most active electrodes of positive voltage were PO8, PO7, P8, and P7. The most active electrodes of negative voltage were CPz and Pz (Figure 7). Thus, the sum and multisensory waveforms between 100 and 140 ms were superimposed for these electrodes specifically. ANOVAs were performed using IBM SPSS^®^Version 25 to determine the impact of group on MSI at this region of greatest neural activity. Statistical analysis was performed separately for the most active electrodes of positive voltage, and the most active electrodes of negative voltage for each of the time bins and the corresponding electrodes (Table 1) using 2 GROUP (control, SCNP) by 2 SIGNAL (SUM, MSI) mixed factors ANOVAs, with repeated measures on the last factor. For all analyses, alpha was set at *p* < 0.05.

MATLAB software R2017a^®^ (Natick, MA, USA) was then used to process the data and to generate graphic representations used to compare differences in the multisensory and sum waveforms between groups at these time points and electrodes. Table 1 demonstrates the electrodes selected based on the scalp distribution at the most active time frames.

#### 2.4.3. EEG/Epochs Analysis Method 2

An adaptation of the Monte Carlo simulation [47] was performed in conjunction with Method 1 in order to further substantiate the results [48]. This method compares the multisensory and sum waveforms in in equal and objective time bins (i.e., 20 ms) to allow for a time course analysis. The electrodes included in this analysis are the same as those used in Method 1, as topographical analysis identified them as having the maximal positive and negative voltage activity during task performance. This method was performed in 20 ms interval time bins for the duration of the peak component, beginning at 60 ms and ending with 180 ms. This was done to capture MSI occurring throughout the entire duration of the maximum multisensory ERP amplitude that occurred during task performance. By investigating the region from 60 to 180 ms, the entire peak is captured, from trough to trough. Figures 6 and 7 clearly represent the greatest amplitude ERPs for both groups of electrodes where significant results were seen.

## 3. Results

### 3.1. Behavioral Data

**Figure 4:**
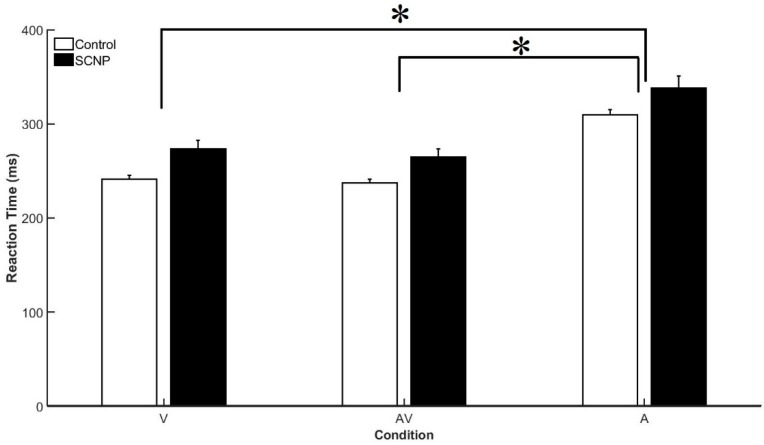
Mean RT for visual (V), auditory (A), and audiovisual (AV) conditions between the control and subclinical neck pain (SCNP) groups. Significant differences (*p* < 0.01) are represented in this graph by the asterisks, as significant mean response times were observed between the AV–A condition and the A–V condition, but not between the V–AV condition.

RT analysis revealed a significant effect of STIMULUS CONDITION, F_2,44_ = 265.25, *p* < 0.001, *η*^2^*p* = 0.923. Both groups demonstrated significantly faster mean RTs to the AV (250.80 ms) and V (257.23 ms) conditions compared to the A (323.97 ms) condition. Further, results present an effect of Group, F_1,22_ = 7.70, *p* < 0.01, *η*^2^*p* = 0.26 demonstrated faster response times for the control group (262.66 ms) compared to the SCNP group (292 ms). Mean RT for all stimulus conditions between both groups are presented in Figure 4.

#### Race Model

**Figure 5:**
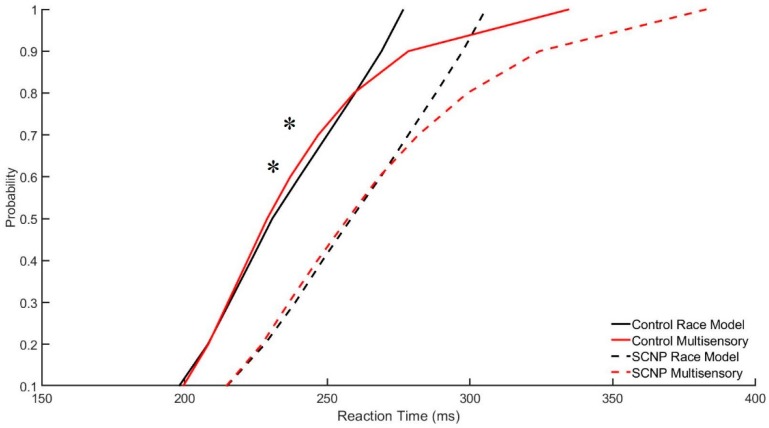
Significant race model violations are represented in this graph by the asterisk, specifically at percent quantiles 0.6 (*p* < 0.05) and 0.7(*p* < 0.03) in the control group.

Paired sample *t*-tests revealed statistically significant results for the control group at the 6th, *t* (11) = −2.39, *p* < 0.05, and 7th, *t* (11) = −2.4, *p* < 0.03, percent quantiles. At these percent quantiles, the multisensory condition probability curve lies to the left of the race model probability curve, indicating that Race Model violations have occurred. This is demonstrated in Figure 5, with the multisensory curve (solid red line) appearing to the left of the race model curve (solid black line). The SCNP group however, does not demonstrate Race Model violations. The SCNP group is represented in Figure 5 with the dashed lines.

### 3.2. Electrophysiological Data

**Figure 6:**
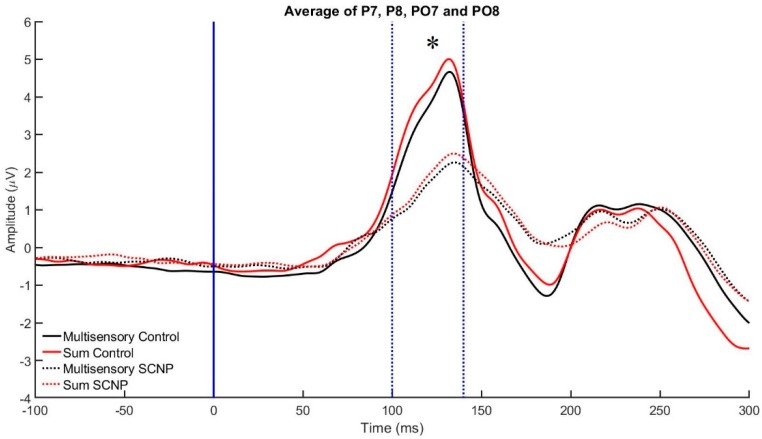
This figure demonstrates the grand average event-related potential (ERP) multisensory integration (MSI) and Sum signals for the control and SCNP groups between 100 and 140 ms at the electrodes od P7, PO7, P8 and PO8. The blue line at 0 ms represents the moment that the stimulus was presented, while the blue lines at 100 ms and 140 ms represent the time bin for which the average brain activity was analyzed. Those without neck pain exhibited greater neural activity levels compared to the SCNP group, as shown by the greater peak amplitudes. The asterisk represents the group effect revealed according to method 1 analysis between 100 and 140 ms (*p* < 0.003).

See Table 1 for the time bins used in this analysis. There were no statistically significant results for the following time bins: 70–90 ms, 150–180 ms, and 180–200 ms post-stimulus presentation. However, at the 100–40 ms time bin for the positive P7, P8, PO7, and PO8 electrodes, an effect of SIGNAL F_1,22_ = 8.753, *p* < 0.007, *η*^2^*p* = 0.294, revealed a significant divergence occurring between the multisensory and sum waveforms, which is indicative of audiovisual integration occurring at these electrodes throughout this 40 ms time period. MSI was therefore occurring during this time frame. A group effect, F_1,22_ = 10.880, *p* < 0.003, *η*^2^*p* = 0.341, indicates that the control group has significantly greater ERP amplitudes than those with SCNP during this 40 ms time period. This group effect is demonstrated in Figure 6. The Monte Carlo time course analysis concurs with these results by also revealing a group effect occurring between 100–120 ms (F_1,22_ = 9.678, *p* < 0.005, partial *η*^2^*p* = 0.315) and 120–140 ms (F_1,22_ = 9.278, *p* < 0.006, *η*^2^*p* = 0.306).

**Figure 7:**
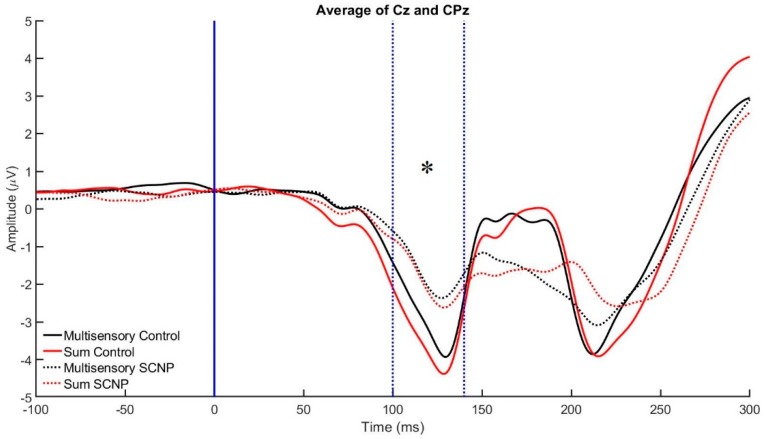
This figure graphically represents differences in the grand average ERP MSI and SUM signals between the control and SCNP groups between 100 and 140 ms at the specified negative voltage electrodes. The control group demonstrates significantly greater neural activity levels, shown by the greater peak amplitudes, as well as significantly greater MSI, shown by greater deviations between the SUM and MSI ERPs. The asterisk represents the group by signal interaction revealed by method one, between the 100 and 140 ms time bin (*p* < 0.005). The control group has a significantly greater divergence between the sum and multisensory waveforms than the SCNP group.

**Figure 8:**
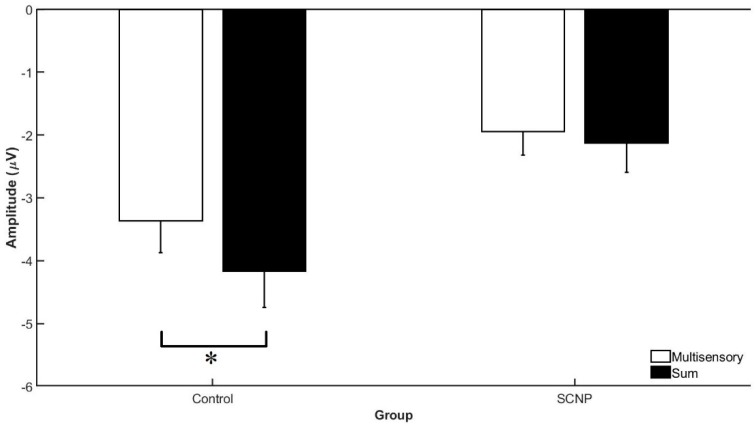
This figure presents group differences in MSI evident at the Cz and CPz electrodes between 100 and 140 ms post-stimulus presentation (*p* < 0.005), as represented by the asterisk. A greater divergence is seen between the MSI and SUM waveforms in the control group in comparison to the SCNP group, which is indicative of a greater degree of multisensory integration.

Based on method 1 analysis, the CPz and Pz electrode activity in the 100–140 ms time bin demonstrates a GROUP by SIGNAL interaction, F_1,22_ = 10.018, *p* < 0.005, *η*^2^*p* = 0.323. This interaction shows that the control group demonstrates a statistically significant greater divergence between the sum and multisensory waveforms than the SCNP group. This indicates that MSI is occurring to a greater extent in the control group compared to their counterparts with SCNP (see Figure 7 and Figure 8). This result is further supported by the results of the Monte Carlo simulation, which demonstrates a GROUP by SIGNAL interaction occurring specifically between the 100–120 ms time bin (F_1,22_ = 5.032, *p* < 0.036, *η*^2^*p* = 0.193), but not between 120–140 ms. In addition, a main effect of GROUP, F_1,22_ = 10.387, *p* < 0.004, *η*^2^*p* = 0.23, shows that the control group has greater brain activity, represented by a greater ERP amplitude, during this time bin compared to the SCNP group (see Figure 8). The Monte Carlo time course analysis reveals a group effect between 100 and 120 ms (F_1,22_ = 5.425, *p* < 0.03, *η*^2^*p* = 0.205), but not between 120 and 140 ms (*p* < 0.083).

## 4. Discussion

This study sought to determine if subclinical neck pain has the potential to lead to differences in the neural processing of A, V, and AV stimuli, demonstrated either by differences in RT or event-related potentials in a neck pain population compared to a healthy population. It should be noted that due to the low number of males in each group, we lacked the statistical power to examine sex differences.

### 4.1. Behavioral Findings

SCNP may lead to alterations in the way that sensory input is transmitted to and processed by the CNS [9]. The results of this study build upon existing research, as in this work, the SCNP group had slower RT for all stimulus conditions as compared to the control group. Although the mean RT for the AV condition was the fastest for both groups, in comparison to the V and A stimuli, the ANOVA did not reveal a significant interaction between the factors of STIMULUS CONDITION andGROUP. Tukey’s post-hoc test revealed that differences between conditions specifically occurred between the V and A and the AV–A conditions, but not between the V–AV condition. These results are in line with previous research that investigated behavioral differences between those with and without neck pain [28]. Given that these results are consistent with the results by Farid et al. (2018), perhaps SCNP has an influence on the processing of afferent visual information. It is interesting that a condition interaction was seen between the A–AV condition in both studies to date investigating long-term influences of neck pain, but no interaction is observed between the V–AV condition. Perhaps participants focused on the V stimulus similarly when presented with the AV condition, as when presented with the unisensory V condition. Moreover, selective attention has an influence on the extent to which MSI occurs and it is possible that this contributed to these results [49]. Although this task has been effectively used in the past by successfully incorporating semantic congruent stimuli presented simultaneously, perhaps future work should incorporate a task of greater complexity to heighten attentional demands [28,30].

The Race Model contributes to these findings, as its violations suggest that differences in RT are due to the processing of multisensory stimuli, rather than being the result of the faster of the two independently processed unisensory stimuli triggering the response. Violations are evident in the control group, while those with SCNP did not show violations, suggesting less MSI in this group. This robust model of human MSI shows that based on the behavioral data, the control group relied on MSI to perform the task, whereas the SCNP group did not. This study shows neural findings in support of this behavioral difference as well.

### 4.2. Electrophysiological Findings

According to the principle of superimposition of electrical fields, a divergence between the multisensory and sum waveforms in a simple RT task is indicative of MSI [23,24,34,46]. In this study, all electrodes analyzed at the 100–140 ms time bin (P7, PO7, P8, PO8, Cz, and CPz) for both groups exhibited this divergence, suggesting that MSI was taking place during task performance in controls and those with SCNP. This study sought to investigate whether differences exist in multisensory integration between a group of individuals with neck pain and those with no previous history of neck pain. The extent of the divergence occurring between these waveforms and differences in the divergence between groups was therefore of interest. The group effect revealed at electrodes P7, PO7, P8, and PO8 using both methods suggests that the control group has a significantly greater ERP amplitude than those with SCNP, 100–140 ms post-stimulus presentation. This means that the overall level of brain activation was higher in the healthy control group in comparison to those with SCNP.

Method 1 results reveal a main effect of GROUP at the Cz and CPz electrodes, between 100–140 ms. This suggests that the control group demonstrated greater levels of brain activity in comparison to the SCNP group. This is represented by the control group’s greater ERP amplitude. The Monte Carlo time course analysis found that this effect was only significant between 100 and 120 ms, and not between 120 and 140 ms. This is likely because the differences are greater in the earlier time bin where the stimulus is approaching its peak processing (highest ERP amplitude). In addition to the main effect of GROUP, Method 1 also reveals a SIGNAL by GROUP interaction between 100–140 ms at these electrodes (*p* < 0.005). Although Method 2 also reveals this interaction (*p* < 0.036), it was only seen between 100–120 ms. The interaction means that at this given time bin, there is a greater divergence occurring between the multisensory and sum waveforms in the control group. The divergence may indicate that MSI is taking place to a greater extent in this group, as supported by the principle of superimposition of electric fields.

This contradicts hypothesis two while supporting hypothesis one, which proposed that due to inverse effectiveness, those with SCNP would potentially demonstrate multisensory enhancement due to greater reliability on multisensory processing. Results support hypothesis one, in which SCNP leads to decrements in multisensory processing. Interestingly, the SCNP population shares this similarity in lack of multisensory and sum waveform differences with the ASD population explored by Brandwein et al. [23]. These authors attribute MSI differences observed in ASD to underlying differences in connectivity. Although SCNP is not a condition like ASD, it has been shown to lead to maladaptive plasticity, changing the function of the prefrontal cortex [50], as well as the cerebellum [13]. It is therefore probable that untreated neck pain could distort appropriate neural integration of various stimulus inputs affecting MSI. An important caveat is that the electrodes selected for each study were based on the grand average of activity for both groups in the study, leading to different electrode selections between the Brandwein study and the current study. This means that the two studies are not directly comparable. It is necessary to consider the brain regions that may have been involved with these changes in multisensory processing. The recognized most active electrodes during the performance of the simple RT multisensory task are, Cz, CPz, P7, PO7, P8 and PO8. Figure 3 demonstrates these electrodes with a 3D head model and assists with capturing the brain regions corresponding to these electrodes. P7, PO7, P8, and PO8 are located in the parietal-occipital lobe of the brain, more specifically in the posterior region along the parietal temporal lobe boarder. The rostral portion of the superior temporal sulcus STS plays an important role in the processing of multisensory stimuli, as it contains a large proportion of multisensory neurons [19]. This structure contains connections to the posterior parietal cortex as well [51]. The current study results are in line with the literature, as they demonstrate heightened neural activity in these regions during performance of the multisensory task. This suggests the involvement of the STS and the posterior parietal cortex in this work, similar to Brandwein and colleagues [23,24], Foxe et al. [34], Molholm et al. [36], and McCracken et al. [33]. In addition to this, the premotor cortex is recognized for its involvement with responses to auditory, visual, and somatosensory inputs [52]. The prefrontal cortex, which is anterior to the premotor cortex, is also known to receive projections from auditory, visual, and multisensory cortical regions, which supports the role of this cortex in multisensory processing [53]. Thus, it is likely that the activity of the Cz and CPz electrodes reflect the activity of the premotor and prefrontal cortices in the current work.

Ultimately, it is important to consider that the similarity between the sum and multisensory waveforms seen in those with neck pain suggests the presence of neurophysiological changes leading to altered processing of sensory information compared to those without SCNP. This supports hypothesis one of this study, as dissimilarity between these ERPs when overlapped was expected. It is also important to note that both groups demonstrated a sum ERP that was of greater amplitude of the multisensory ERP, which is representative of a subadditive multisensory neural response. Multisensory enhancement, however, is still occurring in both groups, as depression and enhancement are both indicators of MSI [19].

Contrary to a previous research conducted by Giard and Peronnet [35], which found evidence of MSI occurring as early as 40 ms, MSI was not evident at such an early time frame in the current study. Onset in this group began at 70 ms, which involves the activity of the posterior auditory cortex. At onset of 40 ms, however, suggests that MSI can occur at about the time that the primary visual cortex is activated. Foxe et al. [34] also demonstrated MSI occurring at an earlier period, but at 50 ms, demonstrating activation in the central/post-central scalp. Although there is evidence of earlier MSI in numerous studies, the current study is not the first to contradict these results. Brandwein et al. [24] demonstrated MSI occurring no earlier than 100 ms. This is in line with the original belief that MSI occurs at periods later than 100 ms, as the sensory input must first be processed [19]. The current literature is inconsistent, perhaps due to differences in the types of populations studied, making identifying the onset of MSI difficult even in healthy and typically developing individuals. Unfortunately, this leads to challenges when comparing results of the current study to a “true baseline”.

Despite this, results of this research do clearly show evidence of multisensory enhancement, due to the differences between the multisensory and sum ERPs in individuals without neck pain compared to those with neck pain. These results obtained, when combined with the behavioral results and Race Model violations of the control group, demonstrate that the extent of multisensory enhancement clearly differs between groups, with the control group demonstrating greater multisensory enhancement, as evidenced by greater dissimilarity between the sum and multisensory waveforms compared to their counterparts who present with neck pain.

## 5. Conclusions

This study presents novel ERP results showing evidence of multisensory differences in an SCNP population. There is limited knowledge on the long-term influence of neck pain on the multisensory system. This study supports previous findings, exemplifying slower RT in those who present with SCNP. This work is the first to use EEG to measure MSI in the SCNP population, and its methods and results may serve as a foundation for future work. A more complex task as used by Farid et al. [28] could be implemented in subsequent research in combination with EEG. Understanding the influence of chronic changes in neck inputon MSI is important neck pain becomes more prevalent due to interactions with technology. Although this study shows that MSI differences may occur as a result of chronic changes in neck sensory input, the mechanism responsible for this is not completely understood and needs further investigation.

## Figures and Tables

**Table 1:** This table presents the most active electrodes during the performance of the audiovisual multisensory task. The time bins were selected based on points in time where brain activity was greatest, as indicated by topographical analysis.

Time Bin (ms)	Positive Voltage (μV)	Negative Voltage (μV)
70–90	N/A	POz, Oz
100–140	P7, PO7, P8, PO8	CPz, Cz
150–150	Oz	N/A
180–200	Oz	N/A
